# High-Throughput Screening of Coenzyme Preference Change of Thermophilic 6-Phosphogluconate Dehydrogenase from NADP^+^ to NAD^+^

**DOI:** 10.1038/srep32644

**Published:** 2016-09-02

**Authors:** Rui Huang, Hui Chen, Chao Zhong, Jae Eung Kim, Yi-Heng Percival Zhang

**Affiliations:** 1 Biological Systems Engineering Department, Virginia Tech, 304 Seitz Hall, Blacksburg, Virginia 24061, USA; 2 Tianjin Institute of Industrial Biotechnology, Chinese Academy of Sciences, 32 West 7th Avenue, Tianjin Airport Economic Area, Tianjin 300308, China

## Abstract

Coenzyme engineering that changes NAD(P) selectivity of redox enzymes is an important tool in metabolic engineering, synthetic biology, and biocatalysis. Here we developed a high throughput screening method to identify mutants of 6-phosphogluconate dehydrogenase (6PGDH) from a thermophilic bacterium *Moorella thermoacetica* with reversed coenzyme selectivity from NADP^**+**^ to NAD^**+**^. Colonies of a 6PGDH mutant library growing on the agar plates were treated by heat to minimize the background noise, that is, the deactivation of intracellular dehydrogenases, degradation of inherent NAD(P)H, and disruption of cell membrane. The melted agarose solution containing a redox dye tetranitroblue tetrazolium (TNBT), phenazine methosulfate (PMS), NAD^**+**^, and 6-phosphogluconate was carefully poured on colonies, forming a second semi-solid layer. More active 6PGDH mutants were examined via an enzyme-linked TNBT-PMS colorimetric assay. Positive mutants were recovered by direct extraction of plasmid from dead cell colonies followed by plasmid transformation into *E. coli* TOP10. By utilizing this double-layer screening method, six positive mutants were obtained from two-round saturation mutagenesis. The best mutant 6PGDH A30D/R31I/T32I exhibited a 4,278-fold reversal of coenzyme selectivity from NADP^**+**^ to NAD^**+**^. This screening method could be widely used to detect numerous redox enzymes, particularly for thermophilic ones, which can generate NAD(P)H reacted with the redox dye TNBT.

Nicotinamide adenine dinucleotide (NAD, which includes NAD^+^ and NADH) and nicotinamide adenine dinucleotide phosphate (NADP, which includes NADP^+^ and NADPH) play distinctive roles in catabolism and anabolism, respectively. NAD and NADP differ in an additional phosphate group esterified at the 2′-hydroxyl group of adenosine monophosphate moiety of NADP (Fig. 1). Numerous redox enzymes use NAD(P) as a coenzyme, which is usually held within the Rossmann fold. Coenzyme engineering that changes coenzyme selectivity (i.e., NAD vs. NADP) of dehydrogenases and reductases is one of the important tools for metabolic engineering and synthetic biology. For example, to produce high-yield biofuels (e.g., butanol, fatty acid esters) under anaerobic conditions, it is essential to balance NADH generation and NAD(P)H consumption^1^,^2^,^3^. Besides the use of transhydrogenase to transfer the hydride from NADH to NADPH^4^,^5^, coenzyme engineering matching coenzyme selectivity of dehydrogenases and reductases is essential to achieve nearly theoretical product yields^6^,^7^,^8^. Coenzyme engineering is also essentially important in biocatalysis. Most times, changing the coenzyme selectivity of dehydrogenases from NADP to NAD is preferable due to (1) NAD is less costly than NADP^9^,^10^ and (2) NADH is more stable than NADPH^11^,^12^,^13^. Also, there are more NADH regeneration enzymes than NADPH regeneration enzymes^14^. Intensive studies have been conducted for changing coenzyme selectivity of dehydrogenases from NADP to NAD^1^,^15^,^16^ and from NAD to NADP^17^,^18^,^19^ as well as broadening coenzyme selectivity^10^. Recent coenzyme engineering studies have expanded the coenzyme selectivity of some redox enzymes to biomimetic coenzymes^9^,^20^,^21^,^22^.

Directed evolution is one of the powerful protein engineering tools that can change enzymes’ substrate selectivity. The most challenging task of directed evolution is the efficient identification of desired mutants from a large mutant library^23^. As for coenzyme engineering, the use of 96-well microplate screening based on the absorbency of NAD(P)H at 340 nm is a straightforward choice^1^. Also, the signal of NAD(P)H can be detected by colorimetric redox indicators. For example, the Arnold’s group utilized a redox dye nitroblue tetrazolium (NBT) plus catalyst phenazine methosulfate (PMS) to determine enhanced thermal stability of 6-phosphogluconate dehydrogenase (6PGDH) with the natural coenzyme (NADP^+^) in the cell lysate of *E. coli*^24^. Later, Zhao and his coworkers applied this method to find out dehydrogenase mutants with relaxed coenzyme selectivity^10^. However, the microplate-based screening is labor-intensive and time-consuming, involving colony picking, liquid cell culture, cell lysis, centrifugation, and enzyme activity assay. Due to high background noise of the intracellular reducing compounds and other redox enzymes in the cell lysate, Banta *et al*. utilized native gels to separate mutants of 2, 5-diketo-D-gluconic acid reductase from the cell lysate, followed by the measurement of UV absorbency changes^25^. However, this method required more steps and had lower capability of screening. Holbrook and his coworkers^26^ developed a method to duplicate colonies from Petri dishes to nitrocellulose paper followed by cell lysis by using lysozyme, detergent, and heat treatment. The targeted dehydrogenase activity was measured by the NBT-PMS assay. Later, Ellington’s group applied this method to identify lactate dehydrogenase mutants with their coenzyme preference change from NAD^+^ to NADP^+ ^27. Nevertheless, this screening method still requires a lot of steps and the throughput is modest due to smearing effect of colony duplication on nitrocellulose paper^26^. Therefore, it is urgently needed to develop a simple and effective high-throughput screening method to determine coenzyme selectivity change of dehydrogenases.

6-phosphogluconate dehydrogenase (6PGDH, EC 1.1.1.44), the third enzyme in the pentose phosphate pathway, converts the 6-phophogluconate and NADP^+^ to ribulose 5-phosphate, NADPH, and CO_2_. 6PGDH from a thermophilic bacterium *Moorella thermoacetica* was utilized to generate NADPH for the high-yield hydrogen production^28^ and generate NADH for electricity generation in biobattery^29^, but the catalytic efficiency (*k*_*cat*_*/K*_*m*_) for NADP^+^ was far higher than that for NAD^+^. Increasing this enzyme’s coenzyme selectivity for NAD^+^ could be important to decrease NADP^+^ use and increase lift-time of biobattery and other applications, such as low-cost biohydrogenation powered by sugars^30^.

In this study, we developed a simple Petri-dish-based double-layer screening for identifying mutants of thermophilic 6PGDH with enhanced catalytic efficiencies for NAD^+^, where the second agarose layer contained a redox dye tetranitroblue tetrazolium (TNBT), a catalyst PMS, 6-phophogluconate, and NAD^+^ and positive mutants were observed by darker color of heat treated colonies. Via this method, several 6GPDH mutants were identified with coenzyme selectivity reversed from NADP^+^ to NAD^+^.

## Results

### Dual promoter plasmid for screening and protein expression

**Figure 2:**
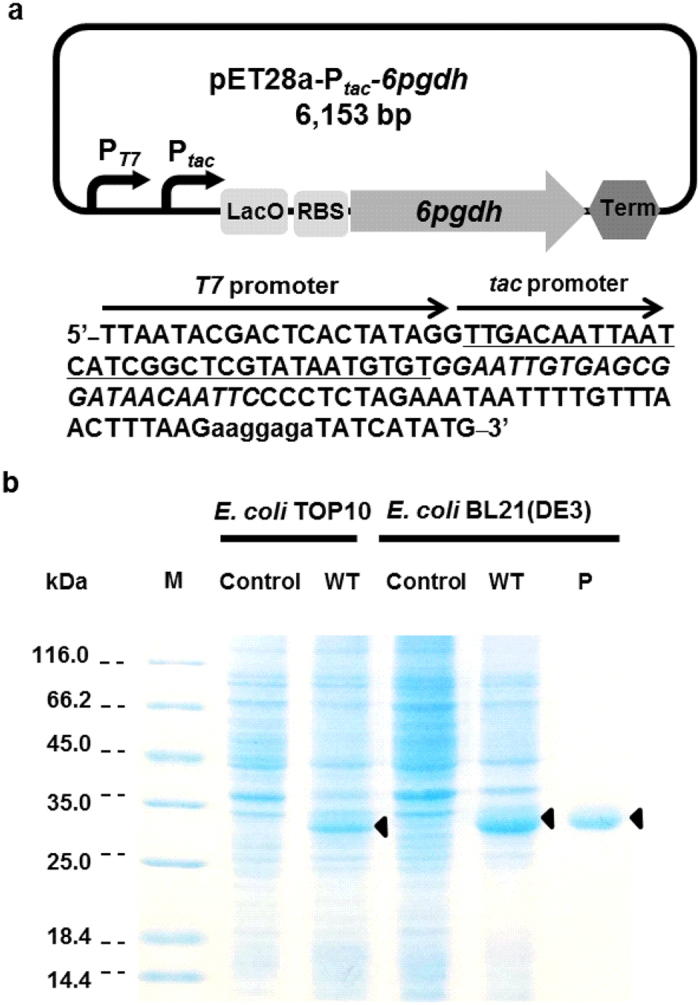
Validation of the dual *T7*-*tac* promoter for 6PGDH screening in *E. coli* TOP10 and protein expression in *E. coli* BL21(DE3). (**a**) The conceptual plasmid map of pET28a-P_*tac*_*-6pgdh*. The DNA sequence of P_*tac*_, lac operator, and RBS were shown as underlined, italic, and small, respectively. (**b**) SDS-PAGE analysis of 6PGDH expression in *E. coli* TOP10 and *E. coli* BL21(DE3). M, protein marker; Control, pET28a-P_*tac*_; WT, pET28a-P_*tac*_*-6pgdh*; P, purified 6PGDH. 6PGDH was indicated with an arrow.

For directed evolution, it is important to create the library with a large number of mutants and express enough recombinant proteins for characterization. In this study, the dual *T7*-*tac* promoter was constructed to control the expression of 6PGDH in both high transformation efficiency host *E. coli* TOP10 and high protein expression host *E. coli* BL21(DE3) (Fig. 2a). Plasmids and strains were listed in Table 1. Plasmid pET28a-P_*tac*_-*6pgdh* consists of a strong inducible promoter *T7*, a modest inducible promoter *tac*, a lac operator, a ribosome binding site (RBS), and the *6pgdh* gene. In *E. coli* TOP10, the modest expression of 6PGDH was accomplished by the *tac* promoter, while the *T7* promoter was inactive due to a lack of T7 RNA polymerase. In *E. coli* BL21(DE3), high expression levels of 6PGDH was obtained under the control of both *T7* and *tac* promoter. As SDS-PAGE analysis showed, although the 6PGDH expression was modest in *E. coli* TOP10, the 6PGDH expression level in *E. coli* BL21(DE3) was high and displayed 4.3-fold greater than that in *E. coli* TOP10 (Fig. 2b).

### Optimization of screening conditions

**Figure 3:**
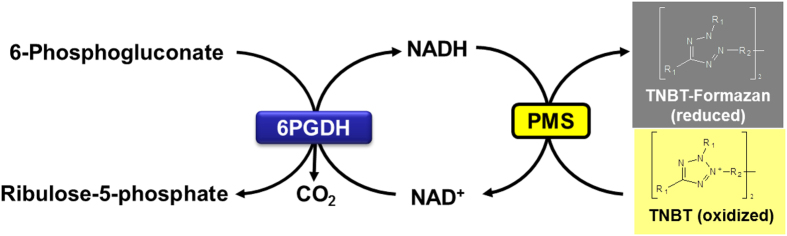
Scheme of the colorimetric assay for 6PGDH activity for NAD^+^. 6PGDH oxidizes 6-phosphogluconate into ribulose-5-phosphate and CO_2_, and reduces NAD^+^ to NADH. In the presence of phenazine methosulphate (PMS) and NADH, the colorless redox dye tetranitroblue tetrazolium (TNBT) was reduced to black TNBT-formazan.

**Figure 4:**
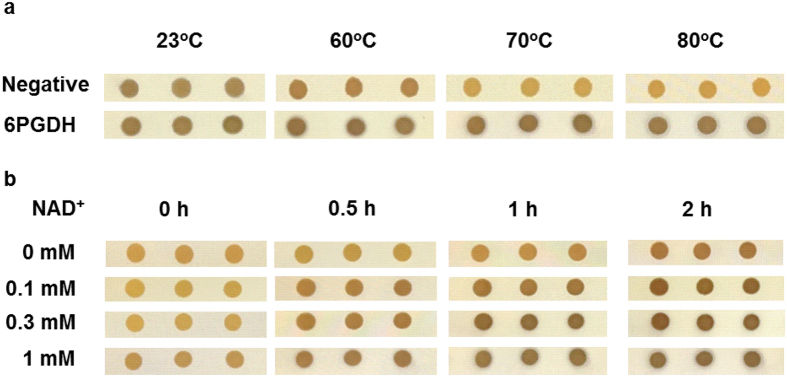
Optimization of heat treatment temperature and color development time. (**a**) Optimization of heat-treatment temperature for screening. *E. coli* TOP10 (pET28a-P_*tac*_) was a negative control while *E. coli* TOP10 (pET28a-P_*tac*_*-6pgdh*) was a positive control. Colonies were treated at 23, 60, 70 and 80 °C for 1 h, respectively. The color changes of the colonies overlaid by the second agarose layer were examined. (**b**) Optimization of color development time and NAD^+^ concentration. Heat-treated colonies of *E. coli* TOP10 (pET28a-P_*tac*_*-6pgdh*) was overlaid by the second layer containing 0, 0.1, 0.3 or 1 mM NAD^+^. The color change profiles of colonies were photographed at 0, 0.5, 1 and 2 h.

The mechanism of colorimetric assay in double-layer screening was shown in Fig. 3. The reduced NADH generated by 6PGDH reacts with TNBT in the presence of PMS, yielding a black TNBT-formazan. Heat-treatment was applied to reduce the background noise from host mesophilic enzymes and metabolites (e.g., NADPH and NADH)^31^ and disrupt cell membranes for NAD^+^ diffusion^32^,^33^. To find out the optimal heat-treatment temperature, two control colonies of *E. coli* TOP10 containing pET28a-P_*tac*_-*6pgdh* (positive) and containing pET28a-P_*tac*_ (negative) were treated at 23, 60, 70 and 80 °C for 1 h. The colonies were then covered by the second layer containing the dye, PMS, NAD^+^, and substrate. As the result showed in Fig. 4a, the positive colonies and the negative colonies without heat treatment developed the same black color. When the heat-treatment temperature was greater than 70 °C, the colonies of the negative control did not develop the black color, indicating the decreased background noises. Positive control colonies expressing 6PGDH exhibited the darker color with haloes regardless of heat-treatment temperatures. Based on the result, the optimal heat-treatment temperature was 70 °C.

The screening conditions were also influenced by NAD^+^ concentration and reaction time. As shown in Fig. 4b, the *E. coli* colonies expressing 6PGDH developed darker color and larger haloes with increasing NAD^+^ concentration and time interval. The colonies with the second layer containing 0 mM NAD^+^ started developing the dark color after 2 h, while *E. coli* TOP10 colonies (pET28a-P_*tac*_) did not develop the color under the same condition (data not shown), implying that the heat-treatment was not enough to degrade *E. coli* NAD(P)^+^ completely^34^,^35^. To minimize the impact of *E. coli* inherent NAD(P)^+^, the screening time was recommended to be less than 2 h.

### Screening 6PGDH mutants for increasing NAD^+^ activity

**Figure 5:**
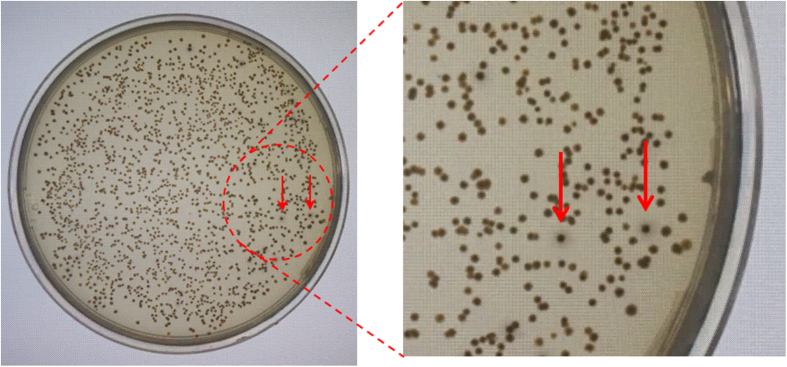
Photo images of the double-layer screening of the mutant library containing two-site mutagenesis of A30/T32. The second layer contained 0.1 mM NAD^+^. The color development time was 1 h. The positive mutants featuring darker colonies with haloes were identified by red arrows.

After optimization of heat-treatment temperature and color development time, the double-layer screening method was used to determine 6PGDH mutants’ coenzyme selectivity change. Figure 5 shows the image of a typical double-layer screening plate containing positive mutants, which were identified by darker color with haloes compared to wild-type and negative mutants. It was found that the color densities of colonies were related to mutant activities for NAD^+^ (data not shown).

To make a reasonable size mutant library with 5-fold coverage, the 6PGDH mutant library was conducted through two-round saturation mutagenesis. In the first round, the site-directed mutagenesis of R31 was conducted and approximately 200 colonies were screened. Two positive mutants, R31T and R31I, were identified and characterized (Table 2). Starting from the best mutant R31I, the two-site-saturated mutagenesis library A30/T32 was constructed. After screening of 5,000 mutants, another four positive mutants, R31I/T32G, A30C/R31I/T32K, A30E/R31I/T32D and A30D/R31I/T32I were identified.

### Characterization of 6PGDH mutants

The activity and kinetic constants for NAD(P)^+^ of wild-type 6PGDH and mutants were summarized in Table 2. Through the first round screening, the R31I had a double *K*_*m*_ value (26.5 μM) for NADP^+^ and a one fourth *K*_*m*_ value (354 μM) for NAD^+^ compared to those of wild-type. Similarly, the R31T exhibited a 3.5-fold reversal due to higher *K*_*m*_ value for NADP^+^ and lower *K*_*m*_ value for NAD^+^. Starting from R31I, the second round mutant R31I/T32G had higher *K*_*m*_ of 104.4 μM for NADP^+^ than that of R31I but no significant change in *K*_*m*_ for NAD^+^. The A30C/R31I/T32K had lower *k*_*cat*_ of 6.23 s^−1^ but much higher *K*_*m*_ of 698 μM on NADP^+^. Also, this mutant had a *k*_*cat*_ value of 6.0 s^−1^ and *K*_*m*_ value of 404 μM on NAD^+^. The A30E/R31I/T32D had a very low *k*_*cat*_ value of 3.1 s^−1^ but a high *K*_*m*_ value of 660 μM for NADP^+^, resulting in catalytic efficiency for NADP^+^ as low as 4.7 mM^−1^ s^−1^. However, the *k*_*cat*_ and K_m_ for NAD^+^ decreased to 10.8 s^−1^ and 127 μM, respectively, resulting in an increase in catalytic efficiency for NAD^+^ to 85.1 mM^−1^ s^−1^.

The best mutant was A30D/R31I/T32I in terms of *k*_*cat*_*/K*_*m*_ for NAD^+^. Comparing with wild-type, the *k*_*cat*_ value for NADP^+^ decreased to 1.81 s^−1^ but the *K*_*m*_ value increased to 228 μM. On the other hand, the *k*_*cat*_ value for NAD^+^ reduced to 5.75 s^−1^ and the *K*_*m*_ value decreased to 11.87 μM, which was comparable to the *K*_*m*_ value of wild-type for NADP^+^ (13.9 μM). The catalytic efficiency of A30D/R31I/T32I for NADP^+^ was decreased by 80-fold, while the catalytic efficiency for NAD^+^ was increased by 54-fold, from 9 to 484.2 mM^−1^ s^−1^, resulting in a 4,278-fold reversal of coenzyme selectivity from NADP^+^ to NAD^+^.

## Discussion

Here we developed an easy high-throughput screening method based on double-layer Petri dishes for determining the coenzyme selectivity of thermophilic 6PGDH for NAD^+^. In this screening, the reduced NADH generated from 6-phosphogluconate catalyzed by 6PGDH mutants could react with TNBT, generating the black TNBT formazan. Although double-layer screening is a very classical enzyme- or microorganism-screening technique without costly instruments, it was surprising that there were few efforts in coenzyme engineering possibly due to multiple reasons. Compared to colony duplication developed by the Holbrook’s group^26^, our method avoided colony duplication and possible smear effects during colony duplication, resulting in less labor and higher throughput screening capacity (e.g., 800 colonies per dish). Furthermore, we applied heat-treatment to kill the *E. coli* cells, disrupt cell membrane^33^,^36^, degrade metabolites including NAD(P)H, and deactivate other *E. coli* enzymes that can work with NAD^+^, but retain intracellular thermophilic 6PGDH for a quick screening. This heat-treatment was efficient to decrease background interference and facilitate substrate mass transfer (Fig. 4) but it also killed the *E. coli* cells, resulting in a problem for recovering *E. coli* cells. To avoid living cell colony replication before heat-treatment as performed previously^23^, we developed an alternative technique to recover the plasmid from dead *E. coli* colonies – picking black dead-cell colonies for micro-plasmid purification followed by the transformation of *E. coli* TOP10. We had a high-throughput screening capacity without any colony replication associated with smear effects and possible cross contamination. As compared to 96- or 384-well plates with assistance of an automatic machine, this high-throughput screening method has several advantages: high screening capacity of approximately 800 colonies per Petri dish, less reagent consumption, less time used for cell cultures and enzyme activity assay, and no need for costly automatic machine that most labs cannot afford.

It was essentially important to find out a suitable redox dye that can react with NADH but have low interference by other compounds. We tested a few redox dyes, including methyl viologen^37^, benzyl viologen^38^, neutral red^39^, methylene blue^40^ and TNBT^41^,^42^. It was found that TNBT was the best because black formazan was very stable in the exposure of air and it had the strongest color change comparing with controls (data not shown). For example, oxidized methylene blue (blue color) is a pH-dependent redox dye that can react with NADH. But its reduced form (colorless) can react with oxygen in air, resulting in slow regeneration of blue color. As a result, this dye was not suitable for screening dehydrogenases whose specific activities were low on non-natural coenzymes.

The *E. coli* TOP10 is a good strain for mutant library construction because of the high transformation efficiencies (e.g., 10^8–9^ cfu/μg plasmid DNA). However, its ability of recombinant protein expression is much lower than the *E. coli* BL21(DE3) utilizing the pET expression system, which suffers from low transformation efficiencies (e.g., 10^6^ cfu/μg plasmid DNA) and possible undesired DNA recombination. A typical directed evolution protocol often involves screening in *E. coli* TOP10 followed by subcloning of mutant’s DNA sequences into pET plasmid and recombinant protein expression in *E. coli* BL21(DE3)^43^,^44^. To delete the subcloning step between screening and protein expression, we developed a dual promoter *T7*-*tac* (Fig. 2a). In *E. coli* TOP10 host growing on the LB medium, the *tac* promoter was responsible for modest expression of the target protein. In *E. coli* BL21(DE3) host plus IPTG, higher protein expression levels were achieved (Fig. 2b).

**Figure 6:**
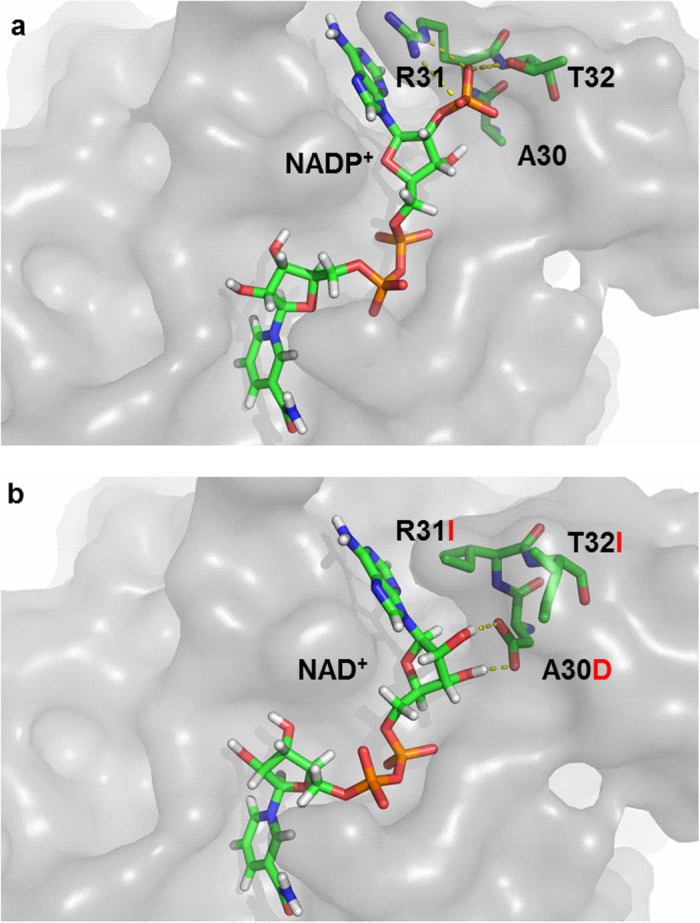
Surface view of wild-type 6PGDH with NADP^+^ (**a**) and mutant A30D/R31I/T32I with NAD^+^ (**b**). The amino acid residues A30, R31 and T32 of wild type 6PGDH and corresponding mutated residues of mutant A30D/R31I/T32I were depicted as sticks and the replacements were marked as red. Atoms were colored according to the types: N, blue; O, red; P, orange; C, green and H, white. Hydrogen bonding between residues and cofactor were shown as yellow line.

Six positive mutants were identified through two round mutant libraries. The arginine at position 31 of wild-type 6PGDH was critical to recognize 2′-phosphate of NADP^+^ and formed double hydrogen bonds with 2′-phosphate by the side chain, which was supported by previous study^45^. Similarly, T32 formed another hydrogen bond with 2′-phosphate through the side chain. Meanwhile, A30 was responsible for the formation of the NADP-binding pocket because of close proximity to 2′-phosphate in the structure model (Fig. 6a). After one-site mutation to isoleucine, the mutant R31I lost the ability of binding the 2′-phosphate of NADP^+^, resulting in a double increase in *K*_*m*_ for NADP^+^ and a four-time decrease in NAD^+^ (Table 2). Similarly, after mutating to threonine, the R31T had a three-fold *K*_*m*_ increase for NADP^+^ but a two-fold *K*_*m*_ decrease for NAD^+^.

The A30D/R31I/T32I was the best mutant in terms of *k*_*cat*_*/K*_*m*_ for NAD^+^. In addition to R31I, the extra mutation of alanine to aspartate at position 30 formed new hydrogen bonds with both 2′ and 3′-hydroxyl group of adenosine monophosphate moiety of NAD^+^ (Fig. 6b) and helped increasing the binding affinity for NAD^+^, further 30-fold decline in *K*_*m*_ for NAD^+^ compared to R31I. The replacement to the other acidic amino acid glutamate at the same position was also found at A30E/R31I/T32D with 3-fold lower *K*_*m*_ for NAD^+^ as compared to R31I. Recently, the mutant included replacement to aspartate at same position was reported for 6PGDH from *G. stearothermophilus* with slightly decreased *K*_*m*_^46^. The mutation threonine to isoleucine at position 32 broke the residual hydrogen bonds with 2′-phosphate of NADP^+^ and possibly decreased enzyme binding with NADP^+^, another 55-fold decrease in catalytic efficiency for NADP^+^. A decrease in binding affinity for NADP^+^ due to a mutation to a hydrophobic amino acid at the same threonine position was also reported for sheep liver 6PGDH mutant T34A^47^. Overall, a combination of the deletion of hydrogen bonds with 2′ phosphate of NADP^+^ at positions 31 and 32 and then addition of more hydrogen bonds with hydroxyl group of NAD^+^ at position 30 resulted in a more than 4,000-fold reversal of coenzyme selectivity from NADP^+^ to NAD^+^.

In conclusion, a high-throughput screening method was established for determining the NAD^+^ selectivity of thermophilic 6PGDH mutants. This double-layer method based on the colorimetric TNBT-PMS assay dramatically decreased dehydrogenase screening labor. The best 6PGDH mutant A30D/R31I/T32I showed a 4,278-fold reversal of coenzyme preference from NADP^+^ to NAD^+^.

## Materials and Methods

### Chemicals, plasmids and strains

All chemicals were reagent grade or higher, purchased from Sigma-Aldrich (St. Louis, MO) or Fisher Scientific (Pittsburgh, PA), unless otherwise noted. The *M. thermoacetica* genomic DNA was purchased from the American Type Culture Collection (Manassas, VA). All enzymes for molecular biology experiments were purchased from New England Biolabs (NEB, Ipswich, MA). Strains, plasmids, and oligonucleotides used in this study are listed in Table 1.

### Construction of pET28a-P_*tac*_-*6pgdh*

Plasmid pET28a-P_*tac*_-*6pgdh* was constructed as follows. The inserted *6pgdh* gene was amplified from *M. thermoacetica* genomic DNA by using a pair of primers 6PG_F/6PG_R and the linearized vector backbone was amplified from pET28a by using a pair of primers 28_back_F/28_back_R. The insertion and vector backbone were assembled into multimeric plasmids by prolonged overlap extension-PCR^48^. The PCR product was directly transformed into *E. coli* TOP10, yielding pET28a-*6pgdh*. To make the dual promoter plasmid pET28a-P_*tac*_-*6pgdh*, the linear backbone of plasmid pET28a-P_*tac*_-*6pgdh* was amplified based on pET28a-*6pgdh* by using a pair of 5′ phosphorylated primers T7_Tac_F/T7_Tac_R containing each half of the promoter P_*tac*_ and was self-ligated by NEB Quick Ligation™ Kit. After transformation into *E. coli* TOP10, plasmid pET28a-P_*tac*_-*6pgdh* was obtained.

### Construction of mutant libraries by saturation mutagenesis

The two-round DNA mutant libraries were constructed by the NEB Phusion site-directed mutagenesis kit. In the first round, the single-site saturation mutagenesis library R31 was amplified based on pET28a-P_*tac*_-*6pgdh* by using a pair of degenerate primers 31_NNK_F/31_NNK_R. The two-site saturation mutagenesis library A30/T32 was amplified from plasmid of pET28a-P_*tac*_-*6pgdh* (R31I) by using a pair of degenerate primers 30_32_NNK_F/30_32_NNK_R. PCR reaction solution (50 μL) containing 1 ng of plasmid template was conducted as follows: 98 °C denaturation for 1 min; 20 cycles of 98 °C denaturation for 30 s, 60 °C annealing for 30 s and 72 °C extension for 3 min; and 72 °C extension for 5 min. The PCR product was digested by *Dpn*I followed by purification of gel electrophoresis and Zymoclean™ Gel DNA Recovery Kit (Zymo Research, Irvine, CA). The purified plasmid library was transformed into *E. coli* TOP10 for screening.

### Optimization of heat treated temperature and time window of HTP screening

In order to find out the optimal heat treated temperature and time window for screening, the *E. coli* TOP10 carrying plasmid pET28a-P_*tac*_(negative control) and *E. coli* TOP10 with pET28a-P_*tac*_-*6pgdh* (positive control) were grown on the 1.5% agar LB medium with 50 μM kanamycin at 37 °C overnight and at room temperature for another day. Colonies of TOP10 (pET28a-P_*tac*_) and TOP10 (pET28a-P_*tac*_-*6pgdh*) were treated at 23, 60, 70 and 80 °C for 1 h, respectively. After cooling down to room temperature, 8 mL of 0.5% (wt/v) melted agarose solution (60 °C) containing 50 mM Tris-HCl (pH 7.5), 50 μM TNBT, 10 μM PMS, 2 mM 6-phosphogluconate, and 1 mM NAD^+^ was carefully poured on the heat-treated colonies. After incubation at room temperature for 1 h, the color change of colonies (to black) was examined on the white background. To find out the right time window for screening, colonies of *E. coli* TOP10 (pET28a-P_*tac*_-*6pgdh*) were treated at 70 °C for 1 h. After cooling down to room temperature, the heat-treated colonies were overlaid by the melted 0.5% (wt/v) agarose solution containing 50 mM Tris-HCl (pH 7.5), 50 μM TNBT, 10 μM PMS, 2 mM 6-phosphogluconate, and a NAD^+^ concentration of 0, 0.1, 0.3 or 1 mM. The color change of different groups was photographed at room temperature at 0, 0.5, 1 and 2 h. Heat-treated colonies had three replicates.

### High-throughput screening of 6PGDH mutants with enhanced NAD^+^ activities

The double-layer screening of 6PGDH mutants on NAD^+^ was performed as follows. After the transformation of the plasmid mutant library, the *E. coli* TOP10 cells were spread on the 1.5% agar LB medium containing 50 μM kanamycin with an expected colony number of 500–800 per Petri dish. The dishes were incubated at 37 °C overnight and at room temperature for another day to ensure enough recombinant 6PGDH expression^49^. The colonies on plates were treated at 70 °C for 1 h to kill cells, deactivate *E. coli* mesophilic enzymes, and degrade metabolites and intracellular coenzymes. Eight mL of 0.5% (wt/v) melted agarose solution (60 °C) containing 50 mM Tris-HCl (pH 7.5), 50 μM TNBT, 10 μM PMS, 2 mM 6-phosphogluconate, and 1 mM (for library R31) or 0.1 mM (for library A30/T32) NAD^+^ was carefully poured on the heat-treated colonies. After incubation at room temperature for 1 h, positive colonies were identified based on the formation of black haloes. The identified colonies embedded in the agarose gel were taken out by a sterile toothstick and then suspended in 200 μL of the P1 buffer of Zymo ZR Plasmid Miniprep™ kit followed by the Zymo ZR plasmid purification protocol. A tiny amount of plasmid extracted from the agarose gel was transformed into *E. coli* TOP10 for plasmid amplification, purification and DNA sequencing.

### Overexpression and purification of wild-type 6PGDH and mutants

Plasmid pET28a-P_*tac*_-*6pgdh* of wild-type or mutants was transformed to *E. coli* BL21(DE3) for protein overexpression in LB medium with 50 μM kanamycin at 37 °C. The protein expression was initiated by adding 0.1 mM IPTG until A_600_ reached ~0.6–0.8. The protein expression was induced at 37 °C for 6 h. Cell pellets were harvested by centrifugation and then were re-suspended in a 20 mM sodium phosphate and 0.3 M NaCl buffer (pH 7.5) containing 10 mM imidazole. After sonication and centrifugation, the supernatant was loaded onto the column packed with HisPur Ni-NTA Resin (Fisher Scientific, Pittsburgh, PA) and then eluted with 20 mM sodium phosphate buffer (pH 7.5) containing 300 mM NaCl buffer and 250 mM imidazole. Mass concentration of protein was determined by the Bradford assay using bovine serum albumin (BSA) as the standard and the 6PGDH expression levels and purified 6PGDH were checked by SDS-PAGE and analyzed by using densitometry analysis of the ImageJ software.

### 6PGDH activity assays

The activities of 6PGDH and mutants were measured in 100 mM HEPES buffer (pH 7.5) with final concentration of 2 mM 6-phosphogluconate, 2 mM NAD(P)^+^, 5 mM MgCl_2_ and 0.5 mM MnCl_2_ at 50 °C for 5 min, as described elsewhere^29^. The formation of NAD(P)H was measured at 340 nm by a UV/visible spectrophotometer (Beckman Coulter, Fullerton, CA). The enzyme unit was defined as one μmole of NAD(P)H produced per min. For determining enzyme kinetic parameters on coenzymes, the enzyme activity was measured in same buffer as described above except changing the concentration of NAD(P)^+^ from 5 to 5000 μM. The result was regressed by GraphPad Prism 5 (Graphpad Software Inc, La Jolla, CA) and apparent *K*_*m*_ and *k*_*cat*_ for NAD(P)^+^ of 6PGDH was given based on Michaelis-Menten nonlinear regression. All the reactions contained three independent replicates and fitted with linear range.

### Structural analysis

The three-dimensional structure modeling of wild-type 6PGDH from *M. thermoacetica* and mutants were built by SWISS-MODEL based on the human 6PGDH (PDB: 2JKV) with 39.4% sequence identity. The structures of NADP^+^ and NAD^+^ were built by using Chemdraw (PerkinElmer, Waltham, MA). Starting from the initial protein and coenzyme structures, the conformation space accessible by NADP^+^ and NAD^+^ binding to the corresponding coenzyme binding area was analyzed by using the Autodock program (Scripps Research Institute, La Jolla, CA).

## Additional Information

**How to cite this article**: Huang, R. *et al*. High-Throughput Screening of Coenzyme Preference Change of Thermophilic 6-Phosphogluconate Dehydrogenase from NADP^+^ to NAD^+^. *Sci. Rep.* **6**, 32644; doi: 10.1038/srep32644 (2016).

## Figures and Tables

**Figure 1:**
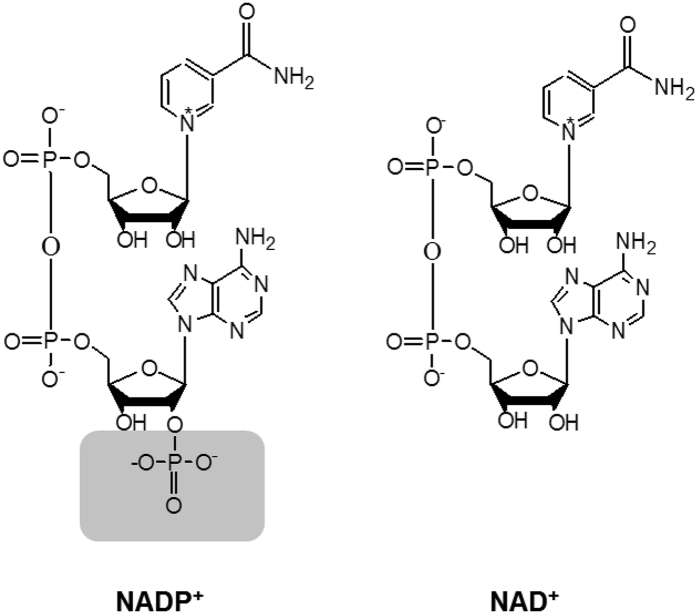
Chemical structures of NADP^+^ and NAD^+^. Structures of NADP^+^ and NAD^+^ were shown and the phosphate group on NADP^+^ was highlighted in gray.

**Table 1:** The strains, plasmids, and oligonucleotides used in this study.

Description	Contents	Reference/sources
**Strain**		
*E. coli* Bl21^star^(DE3)	B F^−^ *ompT gal dcm lon hsdS*_*B*_(*r*_*B*_^−^*m*_*B*_^−^) *rne131* (DE3)	Invitrogen
*E. coli* TOP10	F– *mcr*A cr*mrr*-*hsd*RMS-*mcr*BC) Φ80*lac*Zac80 Δ*lac*X74 *rec*A1 *ara*D139 Δ(*ara leu*) 7697 *gal*U *gal*K *rps*L (StrR) *end*A1 *nup*G	Invitrogen
Plasmid		
pET28a		Invitrogen
pET28a-*6pgdh*	Precusor of pET28a-P_*tac*_*-6pgdh*	in this study
pET28a-P_*tac*_-*6pgdh*	dual promoter (P_*T7*_and P_*tac*_) and moth6pgdh	In this study
primers*		
28_back_F	5′-CGACCAAGACCGACTAAGCCGATATGCATATGTATATCTCCTTCTTAAAG-3′	pET28a
28_back_R	5′-GGCCACGACGTGGCCCGGAAACACCACCACCACCACCACTGAGAT-3′	
6PG_F	5′-CTTTAAGAAGGAGATATACATATGCATATCGGCTTAGTCGGTCTTGGTCG-3′	*Moorella thermoacetica*
6PG_R	5′-ATCTCAGTGGTGGTGGTGGTGGTGTTTCCGGGCCACGTCGTGGCC-3′	
T7_Tac_F	5′-Phos-GGCTCGTATAATGTGTGGAATTGTGAGCGGATAACAATTC-3′	pET28a-*6pgdh*
T7_Tac_R	5′-Phos-GATGATTAATTGTCAACCTATAGTGAGTCGTATTAATTTCG-3′	
31_NNK_F	5′-GAAGTGCGAGGATACGCC**NNK**ACTAAGGCTACCGTGG-3′	pET28a-P_*tac*_-*6pgdh*
31_NNK_R	5′-CCACGGTAGCCTTAGT**MNN**GGCGTATCCTCGCACTTC-3′	
30_32_NNK_F	5′-CATGGTCATGAAGTGCGAGGATAC**NNK**ATT**NNK**AAGGCTACCGTGGACAAAGC-3′	pET28a-P_*tac*_-*6pgdh* R31I
30_32_NNK_R	5′-GCTTTGTCCACGGTAGCCTT**MNN**AAT**MNN**GTATCCTCGCACTTCATGACCATG-3′	

^*^Boldface nucleotide sequences indicate randomized positions.

**Table 2:** Kinetics parameters of 6PGDH and mutants.

Mutations	*K*_*m*_ [μM]		*k*_*cat*_ [s^−1^]		*k*_*cat*_*/K*_*m*_ [mM^−1^ *s^−1^]		Ratio *k*_*cat*_*/K*_*m*_
	NADP^+^	NAD^+^	NADP^+^	NAD^+^	NADP^+^	NAD^+^	NAD^+^/NADP^+^
WT	13. 9 ± 1.1	1397 ± 111	8.73 ± 0.02	12.6 ± 0.4	628.8	9.0	0.014
R31T	35.5 ± 1.7	605 ± 47	10.83 ± 0.13	9.12 ± 0.23	305.6	15	0.049
R31I	26.5 ± 1.3	354 ± 12	11.53 ± 0.15	15.01 ± 0.17	435.0	42.4	0.097
R31I/T32G	104.4 ± 5.4	362 ± 23	11.9 ± 0.16	12.94 ± 0.27	114.0	35.8	0.31
A30C/R31I/T32K	698 ± 49	404 ± 33	6.23 ± 0.18	6.0 ± 0.2	8.9	14.8	1.66
A30E/R31I/T32D	660 ± 64	127 ± 7	3.1 ± 0.1	10.8 ± 0.2	4.7	85.1	18.1
A30D/R31I/T32I	228 ± 16	11.87 ± 0.55	1.81 ± 0.05	5.75 ± 0.05	7.9	484.2	61.1

Each value represents the average of three independent measurements.

## References

[b1] Brinkmann-Chen S. . General approach to reversing ketol-acid reductoisomerase cofactor dependence from NADPH to NADH. Proc. Natl. Acad. Sci. USA 110, 10946–10951 (2013). 23776225 10.1073/pnas.1306073110 PMC3704004

[b2] Huang W.-D. & Zhang Y.-H. P. Analysis of biofuels production from sugar based on three criteria: Thermodynamics, bioenergetics, and product separation. Energy. Environ. Sci. 4, 784–792 (2011).

[b3] Bastian S. . Engineered ketol-acid reductoisomerase and alcohol dehydrogenase enable anaerobic 2-methylpropan-1-ol production at theoretical yield in *Escherichia coli*. Metab. Eng. 13, 345–352 (2011). 21515217 10.1016/j.ymben.2011.02.004

[b4] Hou J., Lages N. F., Oldiges M. & Vemuri G. N. Metabolic impact of redox cofactor perturbations in *Saccharomyces cerevisiae*. Metab. Eng. 11, 253–261 (2009). 19446033 10.1016/j.ymben.2009.05.001

[b5] Gameiro P. A., Laviolette L. A., Kelleher J. K., Iliopoulos O. & Stephanopoulos G. Cofactor balance by nicotinamide nucleotide transhydrogenase (NNT) coordinates reductive carboxylation and glucose catabolism in the tricarboxylic acid (TCA) cycle. J. Biol. Chem. 288, 12967–12977 (2013). 23504317 10.1074/jbc.M112.396796 PMC3642339

[b6] King Z. A. & Feist A. M. Optimal cofactor swapping can increase the theoretical yield for chemical production in *Escherichia coli* and *Saccharomyces cerevisiae*. Metab. Eng. 24, 117–128 (2014). 24831709 10.1016/j.ymben.2014.05.009

[b7] Bommareddy R. R., Chen Z., Rappert S. & Zeng A. P. A *de novo* NADPH generation pathway for improving lysine production of *Corynebacterium glutamicum* by rational design of the coenzyme specificity of glyceraldehyde 3-phosphate dehydrogenase. Metab. Eng. 25, 30–37 (2014). 24953302 10.1016/j.ymben.2014.06.005

[b8] Ehsani M., Fernandez M. R., Biosca J. A. & Dequin S. Reversal of coenzyme specificity of 2,3-butanediol dehydrogenase from *Saccharomyces cerevisae* and *in vivo* functional analysis. Biotechnol. Bioeng. 104, 381–389 (2009). 19507198 10.1002/bit.22391

[b9] Rollin J. A., Tam T. K. & Zhang Y.-H. P. New biotechnology paradigm: cell-free biosystems for biomanufacturing. Green. Chem. 15, 1708–1719 (2013).

[b10] Woodyer R., van der Donk W. A. & Zhao H. Relaxing the nicotinamide cofactor specificity of phosphite dehydrogenase by rational design. Biochemistry 42, 11604–11614 (2003). 14529270 10.1021/bi035018b

[b11] Wu J. T., Wu L. H. & Knight J. A. Stability of NADPH: effect of various factors on the kinetics of degradation. Clin. Chem. 32, 314–319 (1986). 3943190

[b12] Banta S., Swanson B. A., Wu S., Jarnagin A. & Anderson S. Alteration of the specificity of the cofactor-binding pocket of *Corynebacterium* 2,5-diketo-D-gluconic acid reductase A. Protein. Eng. 15, 131–140 (2002). 11917149 10.1093/protein/15.2.131

[b13] Wong C.-H. & Whitesides G. M. Enzyme-catalyzed organic synthesis: NAD (P) H cofactor regeneration by using glucose-6-phosphate and the glucose-6-phosphate dehydrogenase from *Leuconostoc mesenteroides*. J. Am. Chem. Soc. 103, 4890–4899 (1981).

[b14] van der Donk W. A. & Zhao H. Recent developments in pyridine nucleotide regeneration. Curr. Opin. Biotechnol. 14, 421–426 (2003). 12943852 10.1016/s0958-1669(03)00094-6

[b15] Lerchner A., Jarasch A., Meining W., Schiefner A. & Skerra A. Crystallographic analysis and structure-guided engineering of NADPH-dependent *Ralstonia* sp. alcohol dehydrogenase toward NADH cosubstrate specificity. Biotechnol. Bioeng. 110, 2803–2814 (2013). 23686719 10.1002/bit.24956

[b16] Scrutton N. S., Berry A. & Perham R. N. Redesign of the coenzyme specificity of a dehydrogenase by protein engineering. Nature 343, 38–43 (1990). 2296288 10.1038/343038a0

[b17] Hoelsch K., Suhrer I., Heusel M. & Weuster-Botz D. Engineering of formate dehydrogenase: synergistic effect of mutations affecting cofactor specificity and chemical stability. Appl. Microbiol. Biotechnol. 97, 2473–2481 (2013). 22588502 10.1007/s00253-012-4142-9

[b18] Zheng H., Bertwistle D., Sanders D. A. & Palmer D. R. Converting NAD-specific inositol dehydrogenase to an efficient NADP-selective catalyst, with a surprising twist. Biochemistry 52, 5876–5883 (2013). 23952058 10.1021/bi400821s

[b19] Johannes T. W., Woodyer R. D. & Zhao H. Efficient regeneration of NADPH using an engineered phosphite dehydrogenase. Biotechnol. Bioeng. 96, 18–26 (2007). 16948172 10.1002/bit.21168

[b20] Ji D. . Creation of bioorthogonal redox systems depending on nicotinamide flucytosine dinucleotide. J. Am. Chem. Soc. 133, 20857–20862 (2011). 22098020 10.1021/ja2074032

[b21] Paul C. E., Arends I. W. & Hollmann F. Is simpler better? Synthetic nicotinamide cofactor analogues for redox chemistry. ACS Catalysis 4, 788–797 (2014).

[b22] Zhang L., Yuan J., Xu Y., Zhang Y. H. & Qian X. New artificial fluoro-cofactor of hydride transfer with novel fluorescence assay for redox biocatalysis. Chem. Commun. 52, 6471–6474 (2016). 10.1039/c6cc02002j 27100122

[b23] Liu W., Hong J., Bevan D. R. & Zhang Y. H. Fast identification of thermostable beta-glucosidase mutants on cellobiose by a novel combinatorial selection/screening approach. Biotechnol. Bioeng. 103, 1087–1094 (2009). 19388085 10.1002/bit.22340

[b24] Mayer K. M. & Arnold F. H. A colorimetric assay to quantify dehydrogenase activity in crude cell lysates. J. Biomol. Screen. 7, 135–140 (2002). 12006112 10.1177/108705710200700206

[b25] Banta S. & Anderson S. Verification of a novel NADH-binding motif: combinatorial mutagenesis of three amino acids in the cofactor-binding pocket of *Corynebacterium* 2,5-diketo-D-gluconic acid reductase. J. Mol. Evol. 55, 623–631 (2002). 12486521 10.1007/s00239-002-2345-x

[b26] El Hawrani A. S., Sessions R. B., Moreton K. M. & Holbrook J. J. Guided evolution of enzymes with new substrate specificities. J. Mol. Biol. 264, 97–110 (1996). 8950270 10.1006/jmbi.1996.0626

[b27] Flores H. & Ellington A. D. A modified consensus approach to mutagenesis inverts the cofactor specificity of *Bacillus stearothermophilus* lactate dehydrogenase. Protein. Eng. Des. Sel. 18, 369–377 (2005). 16012175 10.1093/protein/gzi043

[b28] Rollin J. A. . High-yield hydrogen production from biomass by *in vitro* metabolic engineering: Mixed sugars coutilization and kinetic modeling. Proc. Natl. Acad. Sci. USA 112, 4964–4969 (2015). 25848015 10.1073/pnas.1417719112 PMC4413329

[b29] Zhu Z., Kin Tam T., Sun F., You C. & Zhang Y.-H. P. A high-energy-density sugar biobattery based on a synthetic enzymatic pathway. Nat. Commun. 5, 3026 (2014). 24445859 10.1038/ncomms4026

[b30] Wang Y., Huang W., Sathitsuksanoh N., Zhu Z. & Zhang Y. H. Biohydrogenation from biomass sugar mediated by *in vitro* synthetic enzymatic pathways. Chem. Biol. 18, 372–380 (2011). 21439482 10.1016/j.chembiol.2010.12.019

[b31] Berridge M. V., Herst P. M. & Tan A. S. Tetrazolium dyes as tools in cell biology: new insights into their cellular reduction. Biotechnol. Annu. Rev. 11, 127–152 (2005). 16216776 10.1016/S1387-2656(05)11004-7

[b32] Zhou Y. . Determining the Extremes of the Cellular NAD(H) Level by Using an *Escherichia coli* NAD^+^ -Auxotrophic Mutant. Appl. Environ. Microbiol. 77, 6133–6140 (2011). 21742902 10.1128/AEM.00630-11 PMC3165392

[b33] Ninh P. H., Honda K., Sakai T., Okano K. & Ohtake H. Assembly and multiple gene expression of thermophilic enzymes in *Escherichia coli* for *in vitro* metabolic engineering. Biotechnol. Bioeng. 112, 189–196 (2015). 25065559 10.1002/bit.25338

[b34] Honda K. . *In vitro* metabolic engineering for the salvage synthesis of NAD^+^. Metab. Eng. 35, 114–120 (2016). 26912312 10.1016/j.ymben.2016.02.005

[b35] Hofmann D., Wirtz A., Santiago-Schübel B., Disko U. & Pohl M. Structure elucidation of the thermal degradation products of the nucleotide cofactors NADH and NADPH by nano-ESI-FTICR-MS and HPLC-MS. Anal. Bioanal. Chem. 398, 2803–2811 (2010). 20803196 10.1007/s00216-010-4111-z

[b36] Ninh P. H. . Development of a continuous bioconversion system using a thermophilic whole-cell biocatalyst. Appl. Environ. Microbiol. 79, 1996–2001 (2013). 23335777 10.1128/AEM.03752-12 PMC3592215

[b37] Do P. M. . Engineering *Escherichia coli* for fermentative dihydrogen production: potential role of NADH-ferredoxin oxidoreductase from the hydrogenosome of anaerobic protozoa. Appl. Biochem. Biotechnol. 153, 21–33 (2009). 19172236 10.1007/s12010-008-8508-5

[b38] Mihara H. . The *iscS* gene is essential for the biosynthesis of 2-selenouridine in tRNA and the selenocysteine-containing formate dehydrogenase H. Proc. Natl. Acad. Sci. USA 99, 6679–6683 (2002). 11997471 10.1073/pnas.102176099 PMC124462

[b39] Park D. H. & Zeikus J. G. Electricity generation in microbial fuel cells using neutral red as an electronophore. Appl. Environ. Microbiol. 66, 1292–1297 (2000). 10742202 10.1128/aem.66.4.1292-1297.2000 PMC91983

[b40] Wilner O. I. . Enzyme cascades activated on topologically programmed DNA scaffolds. Nat. Nano. 4, 249–254 (2009). 10.1038/nnano.2009.50 19350036

[b41] Kugler P. A gel-sandwich technique for the qualitative and quantitative determination of dehydrogenases in the enzyme histochemistry. I. Development of the new methods on the example of LDH (E.C. 1.1.1.27). Histochemistry 60, 265–293 (1979). 468590 10.1007/BF00500656

[b42] Ishizuka H., Tokuoka K., Sasaki T. & Taniguchi H. Purification and some properties of an erythrose reductase from an *Aureobasidium* sp. mutant. Biosci. Biotechnol. Biochem. 56, 941–945 (1992). 27280818 10.1271/bbb.56.941

[b43] Weiß M. S., Pavlidis I. V., Vickers C., Höhne M. & Bornscheuer U. T. Glycine oxidase based high-throughput solid-phase assay for substrate profiling and directed evolution of *(R)*-and *(S)*-selective amine transaminases. Anal. Chem. 86, 11847–11853 (2014). 25321325 10.1021/ac503445y

[b44] Shin H. . Exploring the functional residues in a flavin-binding fluorescent protein using deep mutational scanning. PloS one 9, e97817 (2014). 24887409 10.1371/journal.pone.0097817 PMC4041573

[b45] Tetaud E. . 6-Phosphogluconate dehydrogenase from *Lactococcus lactis*: a role for arginine residues in binding substrate and coenzyme. Biochem. J. 338 **(Pt 1)**, 55–60 (1999). 9931298 PMC1220024

[b46] Opgenorth P. H., Korman T. P. & Bowie J. U. A synthetic biochemistry module for production of bio-based chemicals from glucose. Nat. Chem. Biol. 12, 393–395 (2016). 27065234 10.1038/nchembio.2062

[b47] Li L. & Cook P. F. The 2′-phosphate of NADP is responsible for proper orientation of the nicotinamide ring in the oxidative decarboxylation reaction catalyzed by sheep liver 6-phosphogluconate dehydrogenase. J. Biol. Chem. 281, 36803–36810 (2006). 16959777 10.1074/jbc.M604609200

[b48] You C., Zhang X. Z. & Zhang Y.-H. P. Simple cloning via direct transformation of PCR product (DNA Multimer) to *Escherichia coli* and *Bacillus subtilis*. Appl. Environ. Microbiol. 78, 1593–1595 (2012). 22194286 10.1128/AEM.07105-11 PMC3294473

[b49] Xu J., Banerjee A., Pan S. H. & Li Z. J. Galactose can be an inducer for production of therapeutic proteins by auto-induction using *E. coli* BL21 strains. Protein Expr. Purif. 83, 30–36 (2012). 22425658 10.1016/j.pep.2012.02.014

